# Horseshoe kidney with PLA2R-positive membranous nephropathy

**DOI:** 10.1186/s12882-021-02488-7

**Published:** 2021-08-10

**Authors:** Shuai-Shuai Shi, Xian-Zu Yang, Xiao-Ye Zhang, Hui-Dan Guo, Wen-Feng Wang, Li Zhang, Peng Wu, Wei Zhang, Wen-Bin Wen, Xiao-Lei Huo, Yi-Qiang Zhang

**Affiliations:** 1 Department of Nephrology, Heji Hospital of Changzhi Medical College, Changzhi, 046011 Shanxi China; 2 Graduate School of Changzhi Medical College, Changzhi, 046000 Shanxi China; 3 Department of Imaging, Heji Hospital of Changzhi Medical College, Changzhi, 046011 Shanxi China; 4 Department of Biochemistry, Changzhi Medical College, Shanxi Changzhi, 046000 People’s Republic of China; 5 Department of Jin Yu Renal pathology center, Taiyuan, 030000 Shanxi China; 6 Department of Histology and Embryology, Changzhi Medical College, Changzhi, 046000 Shanxi China

**Keywords:** Horseshoe kidney, Nephritic syndrome, Membranous nephropathy, PLA2R positive

## Abstract

**Background:**

Horseshoe kidney (HSK) is a common congenital defect of the urinary system. The most common complications are urinary tract infection, urinary stones, and hydronephrosis. HSK can be combined with glomerular diseases, but the diagnosis rate of renal biopsy is low due to structural abnormalities. There are only a few reports on HSK with glomerular disease. Here, we have reported a case of PLA2R-positive membranous nephropathy occurring in a patient with HSK.

**Case presentation:**

After admission to the hospital due to oedema of both the lower extremities, the patient was diagnosed with nephrotic syndrome due to abnormal 24-h urine protein (7540 mg) and blood albumin (25 g/L) levels. Abdominal ultrasonography revealed HSK. The patient’s brother had a history of end-stage renal disease due to nephrotic syndrome. Therefore, the patient was diagnosed with PLA2R-positive stage II membranous nephropathy through renal biopsy under abdominal ultrasonography guidance. He was administered adequate prednisone and cyclophosphamide, and after 6 months of treatment, urinary protein excretion levels significantly decreased.

**Conclusion:**

The risk and difficulty of renal biopsy in patients with HSK are increased due to structural abnormalities; however, renal biopsy can be accomplished through precise positioning with abdominal ultrasonography. In the literature, 20 cases of HSK with glomerular disease have been reported thus far. Because of the small number of cases, estimating the incidence rate of glomerular diseases in HSK is impossible, and the correlation between HSK and renal pathology cannot be stated. Further studies should be conducted and cases should be accumulated to elucidate this phenomenon.

## Background

Horseshoe kidney (HSK) is the most common congenital renal fusion malformation that can be diagnosed using imaging techniques such as B-ultrasound and computed tomography (CT) [1]. Patients with HSK can be diagnosed with glomerular disease, and because of the characteristics of the structure of HSK, the risk and difficulty of renal biopsy are relatively high, resulting in fewer pathological results of HSK combined with glomerular disease [2]. This is a case report of HSK complicated by PLA2R-positive membranous nephropathy diagnosed through renal biopsy.

## Case presentation

A 48-year-old male patient was admitted at our hospital because of oedema of both the lower limbs for 1 week. He had no history of hypertension, diabetes, hepatitis, tuberculosis, and drug allergy. The patient’s elder brother was diagnosed with nephropathy in 2008, but he did not undergo renal biopsy. In 2015, the patient was diagnosed with end-stage renal disease and was initiated on haemodialysis.

**Fig. 1:**
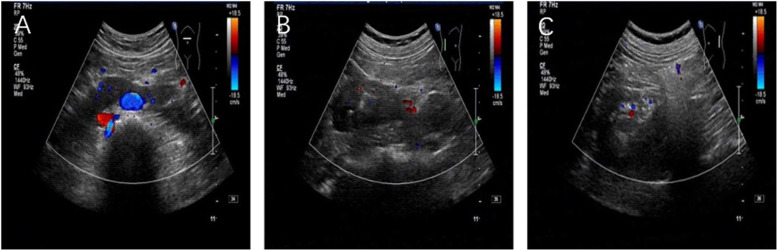
B-mode ultrasonography. **A** The ultrasound probe is placed on the midline of the upper abdomen of the umbilical cord. **B** The ultrasound probe is placed on the right abdomen. **C** The ultrasound probe is placed on the left abdomen

Physical examination on admission showed a blood pressure of 110/70 mmHg, pulse of 85/min, and temperature of 36.5 °C. Abnormalities in the heart, lungs, and abdomen were not observed, but severe oedema on both the lower limbs was noted. Laboratory tests revealed the following results: urine test rendering (++++) proteinuria, (++) haematuria, erythrocyte count of 0–2 cells/high-power field, haemoglobin level of 125 g/dL, total protein level of 45 g/L, serum albumin level of 25 g/L, serum creatinine level of 75 μmol/L, total cholesterol level of 7.8 mmol/L, triglyceride level of 2.5 mmol/L, and serum immunoglobulin and complement levels (C3 and C4) within the normal range. Hepatitis B surface antigen, anti-hepatitis C virus antibodies, and immune deficiency virus antibodies were all negative. The patient’s blood coagulation function was normal. The 24-h urine protein level was 7540 mg. The PLA2R antibody level was 10RU/mL (≤14RU/ml , FICA). Abdominal ultrasonography revealed the presence of HSK (the two kidneys were narrow in shape, the inner structure of the kidney was clear, the urinary collection system was not expanded, and the two kidneys were extremely connected to the front of the abdominal aortic artery and were horseshoe shaped; Fig. 1).

Before the procedure, the patient and his family members signed the informed consent form after being informed of the significance and risks of renal biopsy. Renal biopsy was performed by experienced doctors under ultrasonography guidance at the left renal lower pole using a standard needle biopsy gun. No postoperative complications were observed.

**Fig. 2:**
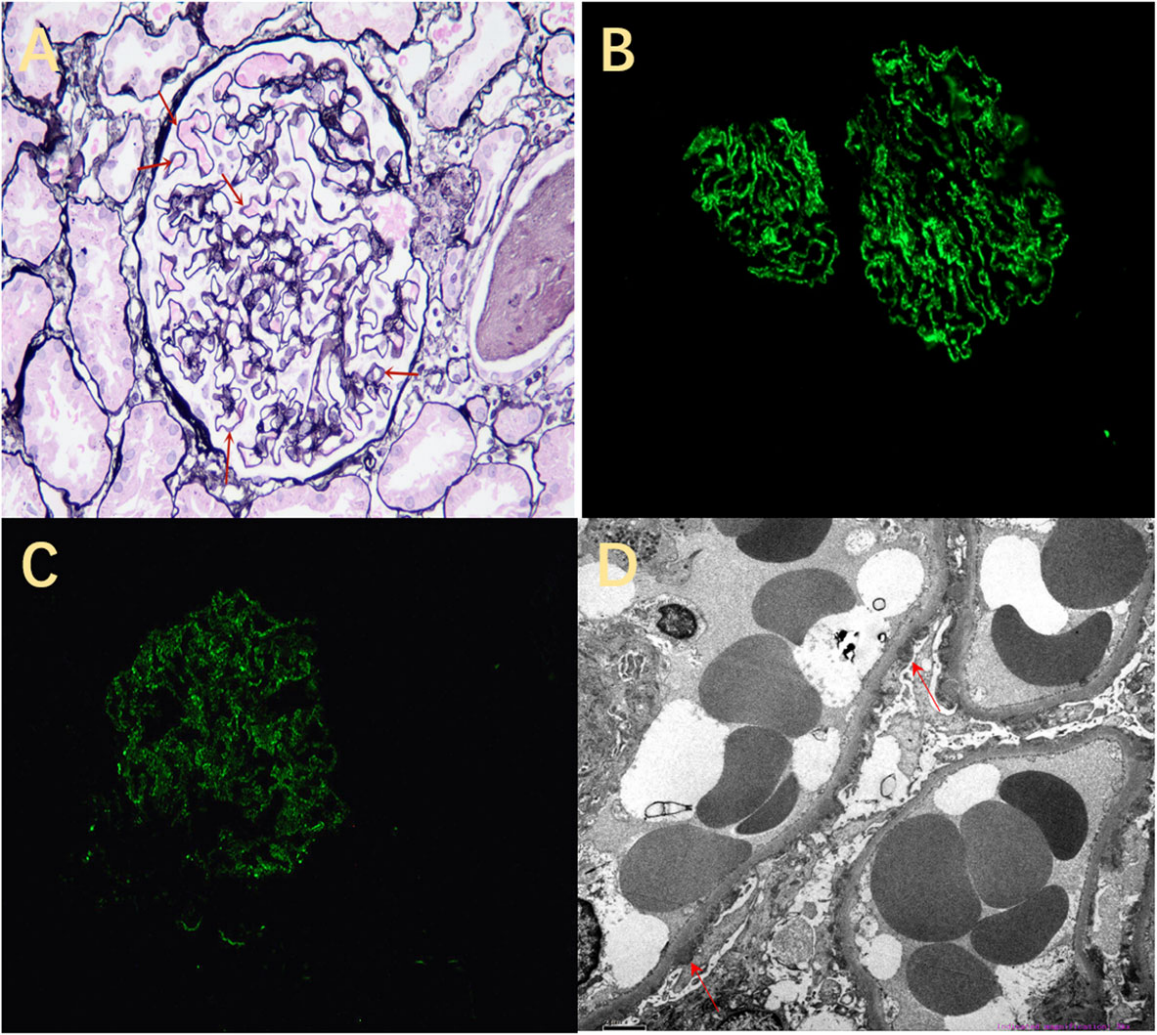
**A** Light micrograph (PASM stain, ×400): The arrow shows basement membrane thickening, nail structure formation; **B** Immunofluorescent stain (× 200): immunoglobulin G4 deposition (3+) in fine granular deposits along the capillary loops. **C** Immunofluorescent stain (× 200): PLA2R (+). (D) Electron microscope (× 3000): The arrow shows the electron dense substance was deposited in the subepithelial and basement membranes of glomerular capillary loops, some spikes

The renal pathological results are shown in Fig. 2. Based on the pathological results of renal puncture, PLA2R-positive membranous nephropathy was diagnosed.

The patient was treated with anticoagulants, angiotensin-converting enzyme inhibitors, hypolipidaemics, prednisone, and cyclophosphamide (CTX). Prednisolone treatment was initiated at 60 mg for 4 weeks and then gradually tapered. CTX was withdrawn after a cumulative dose of 10 g. Proteinuria declined gradually and disappeared over 4 weeks, and oedema disappeared.

After 6 months of treatment, the patient’s 24-h urinary protein level decreased to 110 mg, and the creatinine and albumin levels normalized.

## Discussion and conclusions

Most scholars believe that HSK is an abnormal rotation of the two kidneys during embryonic development [3]. Its incidence rate is approximately 1/600–1/400 [4]. Currently, the diagnosis of HSK is mainly based on abdominal ultrasonography, spiral CT, and nuclear magnetic resonance [5].

Patients with HSK can often have various complications, such as hydronephrosis, urinary calculi, urinary tract infection, other structure-related complications, renal tumours [6, 7], and glomerular disease. Most researchers think that the simultaneous occurrence of HSK and glomerular disease may be a coincidence, but some researchers believe that the abnormal anatomical structure of HSK may be the cause of glomerular disease, which is more likely to lead to long-term chronic repeated stimulation, resulting in antigen–antibody immune complex deposition and renal amyloidosis [8]. However, there is insufficient evidence to elucidate their inevitable causal relationship.

To further clarify the incidence, renal biopsy, pathology, and treatment results of HSK with glomerular disease, case reports and related literature on HSK with glomerular disease were searched, and a total of 20 cases were reviewed (Table 1).

**Table 1:** Domestic and foreign case reports of patients with horseshoe kidney with glomerular disease

Pathological type of renal biopsy	Age (year)	Sex	Therapeutic regimen	Effect of treatment	
MN	20	Male	Prednisolone	Remission	1990 [9]
	48	Female	–	–	1992 [10]
	18	Female	MP + ACEI	Remission	2001 [4]
	–	–	MP + LEF	Remission	2014
FSGS	52	Male	C_S_A	Remission	1991 [11]
	23	Male		–	2007 [8]
		Male	Failure to tolerate therapy	Worsen	2010 [12]
	–	–	MP	Control	2014
IgAN	26	Male	ACEI	Remission	2014 [13]
	–	–	ACEI	Remission	2014
MCD	22	Male	Prednisone	CR	2016 [14]
	64	Female	MP	Remission	2018
M_S_PGN	8	Female	Prednisolone + CTX	Remission	2003
MPGN	38	Male	Prednisone + CTX	Remission	1996
Renal amyloidosis		Male	–	–	2007 [8]
HSPN	15	Female	MP + LEF	Remission	2014
			MP + LEF	CR	2014
LN			MP + MMF	Control	2014
The patient refused (LN)	35	Female	Prednisone + CTX	Remission	2010
No biopsy	27	Female	Prednisone	CR	2016

*MP* Methylprednisolone, *LEF* Leflunomide, *CTX* Cyclophosphamide, *MMF* Mycophenolate mofetil, *CR* Complete remission

Eighteen cases who underwent renal biopsy were diagnosed with membranous proliferative glomerulonephritis(MPGN), renal amyloidosis, Henoch–Schonlein purpuranephritis (HSPN), lupus nephritis (LN), membranous nephropathy (MN), focal segmental glomerulosclerosis (FSGS), mesangial proliferative glomerulonephritis (M_s_PGN), minimal change disease (MCD), and immunoglobulin A nephropathy (IgAN).Two patients who did not undergo renal biopsy were considered to have LN or primary nephrotic syndrome. Our patient with HSK was diagnosed with PLA2R-positive membranous nephropathy through renal biopsy. His elder brother had a history of kidney disease, but not HSK. We believe that this patient may have had familial membranous nephropathy. However, it was difficult to diagnose familial membranous nephropathy owing to insufficient evidence because the patient’s brother did not undergo renal biopsy and the patient did not want to undergo gene testing for familial membranous nephropathy. In the existing literature, there were 4 cases on HSK complicated by membranous nephropathy. But there was no detection of PLA2R antibodies. In this case, the serum level for PLA2R-antibodies was low, and PLA2R was positive in pathology. There was no evidence to prove the relationship between serum PLA2R-antibodies level and horseshoe kidney.

In the litterature the treatment of these patients included glucocorticoids, CTX, and leflunomide. Three of the 20 patients had no treatment-related data. One patient underwent dialysis. The urine protein levels of other patients decreased to varying degrees. In the present case, the patient’s urine protein level was negative by treatment. Combined with the literature, the prognosis of patients with HSK complicated with glomerular disease is mainly related to renal pathology. Therefore, renal biopsy should be performed for subsequent treatment and prognosis, even if the risk of renal biopsy in patients with HSK is high.

The blood supply in HSK varies greatly, and the relationship between the isthmus and blood vessels is complex and variable. These anatomical and vascular abnormalities in HSK make renal biopsy difficult [15]. Before the procedure, patients with HSK need to be carefully evaluated through abdominal ultrasonography, and if necessary, abdominal computed tomography angiography should be performed. In the litterature, the upper part of the left/right kidney was preferred for renal biopsy. The reason may be that 90% of the renal fusion in HSK occurs in the inferior pole, and very few of them occur in the upper pole [4]. In the present case, the middle and lower poles of the left kidney were chosen as the sites of renal biopsy to avoid puncturing the renal upper pole, but fewer glomeruli in the renal specimens were obtained. In our analysis, the small number of glomeruli may be related to the puncture technique and angle, but the abnormal structure of HSK cannot be ruled out.

According to the current case report, it is impossible to confirm the relationship between HSK and glomerular disease, and statistical analysis is needed if further cases are accumulated. However, according to the preliminary analysis, the actual incidence of HSK complicated by glomerular disease may be higher than that reported in the literature, the pathological type is close to the simple pathological type of glomerular disease, and the prognosis is mainly related to the glomerular disease itself. Therefore, when patients with HSK develop glomerular disease, renal biopsy is recommended.

## Data Availability

All data related to this case report are within the manuscript.

## Abbreviations

CR: Complete remission
CT: Computed tomography
CTA: Computer tomography angiography
CTX: Cyclophosphamide
FSGS: Focal segmental glomerulosclerosis
FICA: Fluorescence immunochromatography
HPF: High power field
HSK: Horseshoe kidney
HSPN: Henoch-Schoenlein purpura nephritis
IgAN: IgA nephropathy
LEF: Leflunomide
LN: Lupus nephritis
MCD: Minimal change disease
MMF: Mycophenolate mofetil
MN: Membranous nephropathy
MP: Methylprednisolone
MPGN: Membranous proliferative glomerulonephritis
MsPGN: Mesangial proliferative glomerulonephritis
PLA2R: Phospholipase A2 receptor
